# Neutrophil to Lymphocyte Ratio in Maternal Blood: A Clue to Suspect Amnionitis

**DOI:** 10.3390/jcm10122673

**Published:** 2021-06-17

**Authors:** Joon-Hyung Lee, Chan-Wook Park, Kyung-Chul Moon, Joong-Shin Park, Jong-Kwan Jun

**Affiliations:** 1 Department of Obstetrics and Gynecology, Seoul National University College of Medicine, Seoul 03080, Korea; kontractubex12@gmail.com (J.-H.L.); jsparkmd@snu.ac.kr (J.-S.P.); jhs0927@snu.ac.kr (J.-K.J.); 2 Institute of Reproductive Medicine and Population, Seoul National University Medical Research Center, Seoul 03080, Korea; 3 Department of Pathology, Seoul National University College of Medicine, Seoul 03080, Korea; blue7270@gmail.com

**Keywords:** amnionitis, maternal blood, neutrophil to lymphocyte ratio, preterm birth

## Abstract

There is no information about whether maternal neutrophil to lymphocyte ratios (NLRs) progressively increase with respect to the progression of acute histologic chorioamnionitis (acute-HCA) and increased maternal NLR is a risk factor for amnionitis, known as advanced acute-HCA, in pregnant women at risk for spontaneous preterm birth (PTB). The objective of the current study is to examine this issue. The study population included 132 singleton PTB (<34 weeks) due to either preterm labor or preterm-PROM with both placental pathology and maternal CBC results within 48 h before delivery. We examined maternal NLRs according to the progression of acute-HCA in extra-placental membranes (EPM) (i.e., group-0, inflammation-free EPM; group-1, inflammation restricted to decidua; group-2, inflammation restricted to the membranous trophoblast of chorion and the decidua; group-3, inflammation in the connective tissue of chorion but not amnion; group-4, amnionitis). Maternal NLRs significantly and progressively increased with the progression of acute-HCA (Spearman’s rank correlation test, γ = 0.363, *p* = 0.000019). Moreover, the increased maternal NLR (≥7.75) (Odds-ratio 5.56, 95% confidence-interval 1.26-24.62, *p* < 0.05) was a significant independent risk factor for amnionitis even after the correction for potential confounders. In conclusion, maternal NLRs significantly and progressively increased according to the progression of acute-HCA and the increased maternal NLR (≥7.75) was an independent risk factor for amnionitis in spontaneous PTB. The evaluation of the performance of NLR should clearly require a prospective description of this parameter in a cohort of patients with either threatened PTL or preterm-PROM.

## 1. Introduction

Ascending intrauterine infection is one of the major physiologies in spontaneous preterm birth (PTB) (i.e., preterm labor and intact membranes (PTL) and preterm premature rupture of membranes (preterm-PROM)) [1,2]. Micro-organisms from the vaginal and cervical canal ascend to chorio-decidua and advance to the amnion in extra-placental membranes (EPM) [2]; this eventually results in fetal infection [1,2,3]. During the progression of ascending intrauterine infection, maternal neutrophils sequentially migrate from the decidua through the membranous trophoblast of chorion to the connective tissue of chorion and finally infiltrates into amnion in EPM [4]. Acute histologic chorioamnionitis (acute-HCA) generated by neutrophils infiltration into the EPM is considered a maternal inflammatory response because neutrophils in EPM are derived from maternal vessels of decidua parietalis [5,6].

It is well known that intra-amniotic inflammatory responses are closely associated with acute-HCA [7,8,9,10]. Moreover, our previous study reported that intra-amniotic inflammatory responses increase with outside-in neutrophils migration in the chorio-decidua (i.e., ‘inflammation restricted to decidua’, ‘inflammation restricted to the membranous trophoblast of chorion’, and ‘inflammation in the connective tissue of chorion’) [11]. Moreover, intra-amniotic and fetal inflammatory responses are more intense and the early-onset neonatal sepsis is more frequent in amnionitis (more advanced stage inflammation) than in inflammation restricted to chorio-decidua (less advanced stage inflammation) of EPM [12]. In general, intra-amniotic inflammatory response is gauged by several markers (i.e., white blood cell (WBC) count, matrix metalloproteinase-8 (MMP-8), and IL-6 in amniotic fluid (AF) obtained by amniocentesis. However, amniocentesis is an invasive procedure and may not be feasible in cases with decreased AF volume in the context of preterm-PROM. Therefore, numerous studies attempted to find potential markers in maternal blood but not in AF for the identification of acute-HCA in EPM (Table S1) [13,14,15,16,17,18,19,20,21,22,23,24,25,26,27,28,29,30,31,32,33,34,35,36,37,38,39,40,41,42,43,44,45,46]. However, acute-HCA remains unpredictable with the use of maternal inflammatory blood markers and, moreover, there are limitations in previous studies as follows (Table S1); (1) no previous studies examined maternal inflammatory blood markers according to the progression of acute-HCA in the sub-divisions of EPM (i.e., decidua, the membranous trophoblast of chorion, the connective tissue of chorion, and amnion) [13,14,15,16,17,18,19,20,21,22,23,24,25,26,27,28,29,30,31,32,33,34,35,36,37,38,39,40,41,42]; (2) a substantial number of studies did not adjust for gestational age (GA) at delivery or maternal blood sampling [13,14,16,17,18,19,20,23,24,25,26,27,28,30,31,32,33,34,35,36,37,38,39,40,41,42] and did not provide a meaningful temporal relationship between the maternal inflammatory blood tests and the placental pathologic examinations after delivery [16,17,22,23,24,25,27,31,32,41].

Recently, the neutrophil to lymphocyte ratio (NLR) as a biomarker for systemic inflammatory conditions in adults is known to be positively correlated with disease activity in rheumatic disease [47,48,49,50,51,52,53] and known to be associated with the prognosis (i.e., survival) of sepsis, systemic inflammatory response syndrome (SIRS), and septic shock [54,55,56,57,58] in patients. Moreover, some researchers demonstrated that increased neonatal NLR is a marker or predictor for significant neonatal morbidities (i.e., early-onset neonatal sepsis [EONS], broncho-pulmonary dysplasia (BPD), and necrotizing enterocolitis (NEC)) [59,60,61]. What is noteworthy is that maternal NLRs are reported to be elevated in cases with preeclampsia [62,63,64], which is associated with exaggerated inflammatory responses in the maternal vascular system [65]. However, there is no information on the relationship between maternal NLRs and the progression of acute-HCA among pregnant women at risk for PTB in the current body of research. We hypothesized that maternal NLRs progressively increase according to the progression of acute-HCA and increased maternal NLR is a risk factor for amnionitis known as advanced acute-HCA among pregnant women at risk for spontaneous PTB. We additionally examined maternal high-sensitivity C-reactive protein (hs-CRP) concentrations to demonstrate the usefulness of maternal NLR for the identification of amnionitis. The objective of the current study is to examine this issue.

## 2. Materials and Methods

### 2.1. Study Design and Patient Population

The study population included 132 singleton pregnant women who met the following criteria: (1) Korean; (2) GA at delivery between 20.6 weeks and 33.9 weeks; (3) PTB due to either PTL (63 cases) or preterm-PROM (69 cases); (4) available placental pathologic slides; (5) maternal complete blood count (CBC) profile available within 48 h before delivery. The last criterion was used to preserve a meaningful temporal relationship between maternal CBC profiles and placental pathologic findings at delivery. At our institution, the maternal CBC test and placental pathologic examination after delivery were routinely recommended and performed to all pregnant women hospitalized with either PTL or preterm-PROM. PTL and preterm-PROM were diagnosed in accordance with previously published criteria [8,9]. Written informed consent was obtained from the entire study population. The Institutional Review Board of our institute specifically approved the current study.

### 2.2. Clinical Characteristics and Pregnancy Outcomes

Clinical characteristics and pregnancy outcomes were investigated from medical records. Data included maternal age, parity, clinical history of antenatal vaginal bleeding or the evidence of placenta previa, cause of preterm delivery, gender of newborn, delivery mode, GA at delivery, birth weight, 1 min and 5 min Apgar scores, meconium staining, antenatal use of corticosteroids, antenatal use of antibiotics, and antenatal use of tocolytics.

### 2.3. Diagnosis of Acute Histologic Chorioamnionitis (Acute-HCA) in Extra-Placental Membranes (EPM)

Placental tissue samples for pathologic examination included EPM (i.e., chorio-decidua and amnion), chorionic plate, and the umbilical cord. These samples were fixed in 10% neutral buffered formalin and embedded in paraffin. Sections of prepared tissue blocks were stained with hematoxylin and eosin (H&E). Clinical information regarding the placental tissues was not disclosed to pathologists. Acute-HCA in EPM was defined as the presence of neutrophil infiltration in either chorio-decidua or amnion. Acute inflammation in chorio-decidua and amnion was diagnosed according to the previously published criteria: (1) Chorio-deciduitis was diagnosed in the presence of at least one focus of >5 neutrophils in chorio-decidua; (2) amnionitis was diagnosed in the presence of at least one focus of >5 neutrophils in amnion. The progression of acute-HCA in EPM was divided according to outside-in neutrophils migration in EPM as follows: (1) group-0, inflammation-free EPM; (2) group-1, inflammation restricted to decidua; (3) group-2, inflammation restricted to the membranous trophoblast of chorion and the decidua; (4) group-3, inflammation in the connective tissue of chorion but not amnion; (5) group-4, amnionitis.

### 2.4. Maternal Neutrophil to Lymphocyte Ratio (NLR)

Maternal blood was collected in ethylenediaminetetraacetic-acid (EDTA) tubes by venipuncture of the antecubital vein within 48 h before delivery and CBC with differential leukocyte count was performed. NLR is defined as absolute neutrophil count divided by absolute lymphocyte count. We additionally examined maternal hs-CRP concentrations within 48 h before delivery to demonstrate the usefulness of maternal NLR for the identification of amnionitis.

### 2.5. Statistical Analysis

Continuous and categorical variables were compared with the Kruskal–Wallis test and Pearson’s chi-square test, respectively. Multiple comparisons of continuous and categorical variables between the groups according to the progression of acute-HCA in EPM were performed with 1-way ANOVA with post-hoc Tukey test and Fisher’s exact test with Bonferroni’s correction, respectively. Spearman’s rank correlation test was used to examine the relationship between maternal NLRs and acute-HCA in EPM. The receiver operating characteristics (ROC) curve was used to estimate the best cut-off values (maximum sum of sensitivity and specificity) and to identify maternal NLRs as being raised or not raised for the detection of amnionitis. Using this cut-off value, we compared the frequency of increased maternal NLR according to the progression of acute-HCA in EPM with Pearson’s chi-square test. Moreover, linear by linear association was used to investigate the trend about the frequency of increased maternal NLR (≥7.75) according to the progression of acute-HCA in EPM. Diagnostics indices (i.e., sensitivity, specificity, positive predictive value, negative predictive value, positive likelihood ratio, and negative likelihood ratio) were determined for increased maternal NLR for the identification of amnionitis. We performed multiple logistic regression analysis for the exploration of the relationship between various variables and amnionitis. We analyzed maternal hs-CRP with the same statistical methods to demonstrate the usefulness of maternal NLR for the identification of amnionitis. Statistical significance was defined as *p* < 0.05.

## 3. Results

### 3.1. Clinical Characteristics and Pregnancy Outcomes According to the Progression of Acute Histologic Chorioamnionitis (Acute-HCA) in Extra-Placental Membranes (EPM)

Group-0, group-1, group-2, group-3, and group-4 was present in 36.4% (48/132), 14.4% (19/132), 20.5% (27/132), 17.4% (23/132), and 11.4% (15/132) of study population, respectively (Table 1). Table 2 demonstrated that GA at delivery and birth weight were significantly decreased according to the progression of acute-HCA in EPM and there was a significant difference in the frequency of antenatal use of antibiotics among five groups according to the progression of acute-HCA in EPM (Table 2).

### 3.2. Maternal Neutrophil to Lymphocyte Ratios (NLRs) According to the Progression of Acute Histologic Chorioamnionitis (Acute-HCA) in Extra-Placental Membranes (EPM)

**Figure 1:**
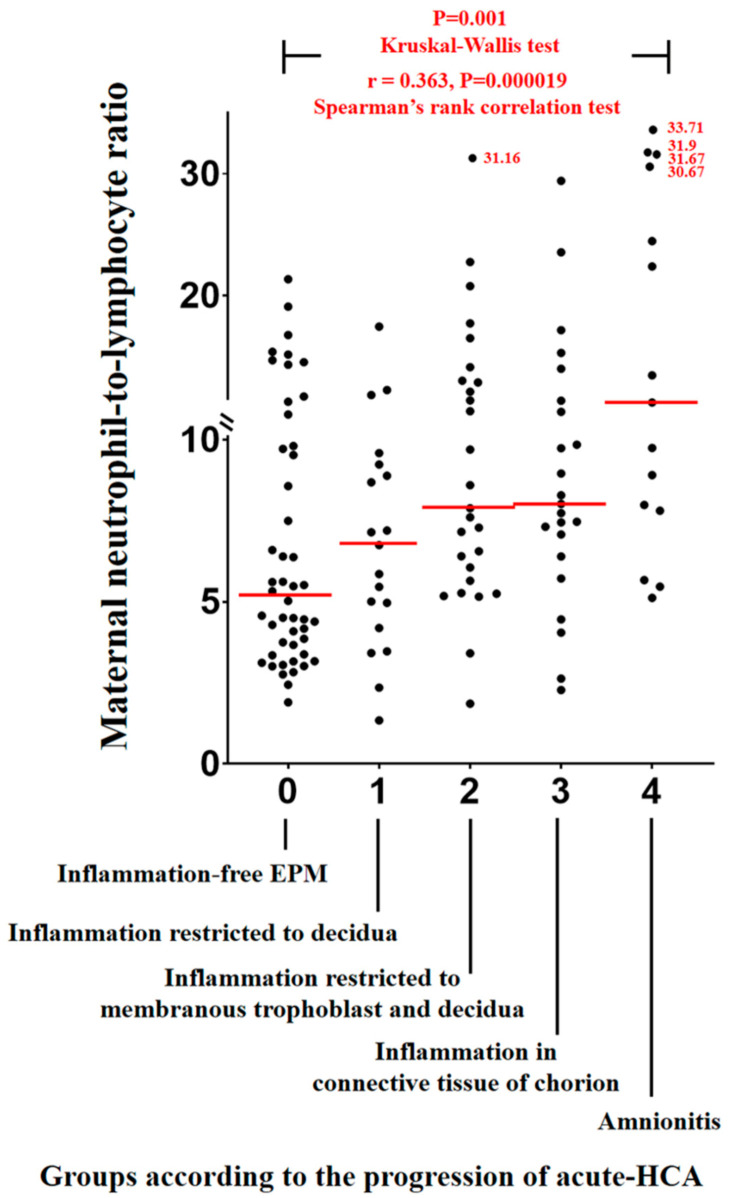
Maternal neutrophil to lymphocyte ratios (NLRs) according to the progression of acute histologic chorioamnionitis (acute-HCA) in extra-placental membranes (EPM). Maternal NLRs significantly and progressively increased with the progression of acute-HCA (group-0 vs. group-1 vs. group-2 vs. group-3 vs. group-4; median, range; 5.15 (1.90–21.30) vs. 6.70 (1.30–17.40) vs. 7.90 (1.90–31.20) vs. 8.00 (2.30–29.40) vs. 11.20 (5.10–33.70)). Each *p* value is shown in the graph.

Figure 1 shows maternal NLRs according to the progression of acute-HCA in EPM. Maternal NLRs significantly and progressively increased with the progression of acute-HCA (Kruskal–Wallis test, *p* = 0.001; and Spearman’s rank correlation test, γ = 0.363, *p* = 0.000019) (Figure 1). Maternal hs-CRP (mg/dL) also significantly and progressively increased with the progression of acute-HCA (Kruskal–Wallis test, *p* = 0.006; and Spearman’s rank correlation test, γ = 0.298, *p* = 0.000900) (Figure S1).

### 3.3. Diagnostic Indices, Predictive Values, and Likelihood Ratios of Increased Maternal Neutrophil to Lymphocyte Ratio (NLR) for the Identification of Amnionitis

ROC curves were constructed to select the cut-off values for identifying maternal NLR (area under curve (AUC), 0.745; standard error (SE), 0.066; *p* = 0.002) as being raised or not raised for the identification of amnionitis and a cut-off value of 7.75 was chosen (Figure S2, red line). Moreover, for the comparison with maternal NLR, we constructed a ROC curve to choose the cut-off values for the discovery of maternal hs-CRP (AUC, 0.581; SE, 0.086; *p* = 0.323) as being raised or not raised for the diagnosis of amnionitis and a cut-off value of 1.035 mg/dL was chosen (Figure S2, blue line). Table 2 displays diagnostic indices, predictive values, and the likelihood ratios of increased maternal NLR (≥7.75) within 48 h before delivery for the identification of amnionitis. Moreover, we demonstrated diagnostic indices, predictive values, and likelihood ratios of maternal hs-CRP ≥ 1.035 mg/dL within 48 h before delivery for the identification of amnionitis in cases with either PTL or preterm-PROM (Table S2). However, these positive and negative likelihood ratios were not significant (Table S2).

### 3.4. The Frequency of Increased Maternal Neutrophil to Lymphocyte Ratio (NLR) According to the Progression of Acute Histologic Chorioamnionitis (Acute-HCA) in Extra-Placental Membranes (EPM)

**Figure 2:**
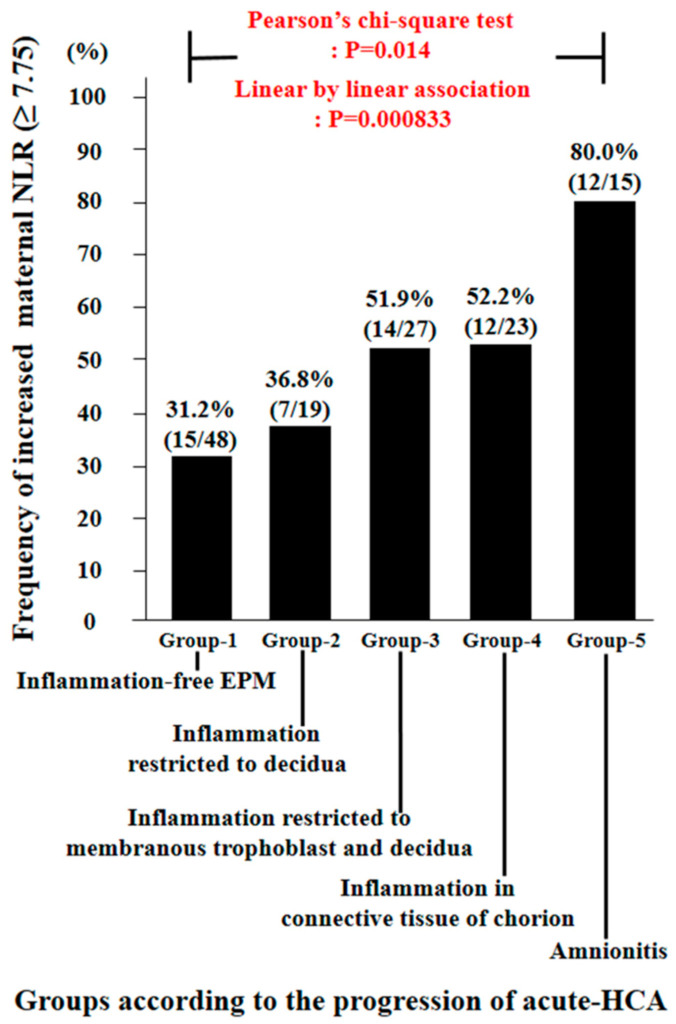
Frequency of increased maternal neutrophil to lymphocyte ratio (NLR) (≥7.75) according to the progression of acute histologic chorioamnionitis (acute-HCA) in extra-placental membranes (EPM). Each *p* value is shown in the graph.

There was a significant stepwise increase in the frequency of increased maternal NLR (≥7.75) according to the progression of acute-HCA in EPM (Pearson’s chi-square test, *p* = 0.014; and linear by linear association, *p* = 0.000833) (Figure 2). Moreover, Table 3 demonstrated that increased maternal NLR (≥7.75) was a significant independent risk factor for amnionitis even after the correction for potential confounding variables. We additionally demonstrated the frequency of increased maternal hs-CRP (≥1.035 mg/dL) according to the progression of acute-HCA in EPM (Figure S3). However, increased maternal hs-CRP ≥ 1.013 mg/dL was not an independent risk factor for amnionitis (Table S3).

### 3.5. Histopathology and Schema of the Progression of Acute Histologic Chorioamnionitis (Acute-HCA) in Extra-Placental Membranes (EPM)

**Figure 3:**
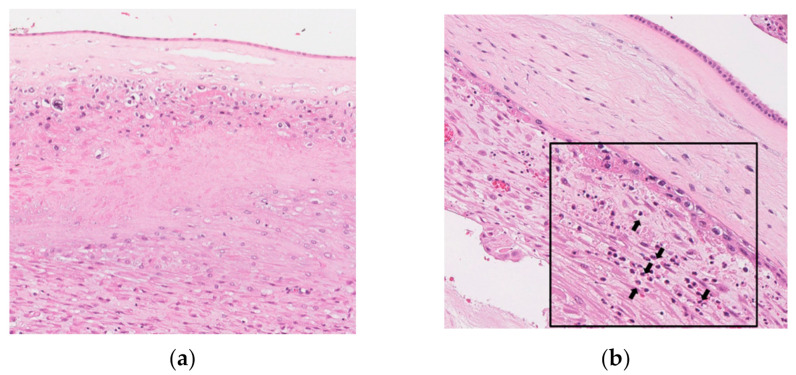

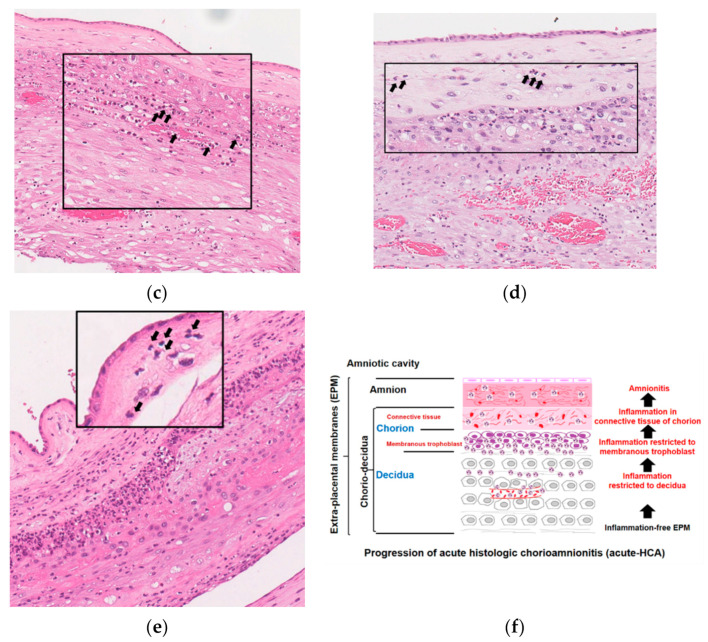
Histopathology and schema of the progression of acute histologic chorioamnionitis (acute-HCA). Hematoxylin and eosin stained histologic sections of extra-placental membrane (EPM) are shown as follows: (**a**) group-0, inflammation-free EPM; (**b**) group-1, inflammation restricted to decidua; (**c**) group-2, inflammation restricted to the membranous trophoblast of chorion and the decidua; (**d**) group-3, inflammation in the connective tissue of chorion but not amnion; (**e**) group-4, amnionitis. These images are based on the magnification setting ×200 and the insets of panels are based on the magnification setting ×400. Some neutrophils are shown in the decidua (group-1) (**b**), the membrane trophoblast of chorion (group-2) (**c**), the connective tissue of chorion (**d**), and amnion (**e**) (see the insets of panels). Black arrows in the insets of panels indicate neutrophils infiltrating into EPM (**b**–**e**). The schema of the progression of acute-HCA depicts outside-in neutrophils migration in the whole sub-divisions of EPM (**f**).

Figure 3 shows representative images for inflammation-free EPM (a, group-0), inflammation restricted to decidua (b, group-1), inflammation restricted to the membranous trophoblast of chorion and the decidua (c, group-2), inflammation in the connective tissue of chorion but not amnion (d, group-3), and amnionitis (e, group-4) in H&E stained histologic sections of EPM. Figure 3f is the schema depicting the progression of acute-HCA generated by outside-in neutrophils migration in the entire sub-divisions of EPM.

## 4. Discussion

### 4.1. Principal Findings of This Study

**Figure 4:**
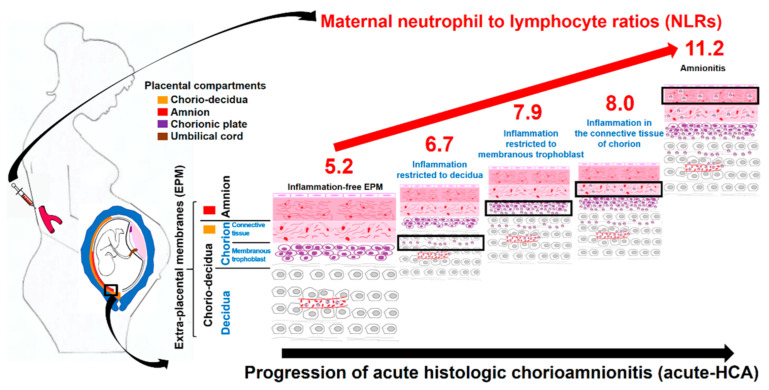
Schema of maternal neutrophil to lymphocyte ratios (NLRs) according to the progression of acute histologic chorioamnionitis (acute-HCA).

Maternal NLRs significantly and progressively increased according to the progression of acute-HCA (Figure 4) and increased maternal NLR (≥7.75) was an independent risk factor for amnionitis in spontaneous PTB. This finding suggests maternal NLR may be used as a non-invasive antenatal marker for amnionitis.

### 4.2. Limitations of Previous Studies Reporting the Relationship between Maternal Inflammatory Blood Markers and Acute Histologic Chorioamnionitis (Acute-HCA) in Extra-Placental Membranes (EPM)

There is a good chance that maternal inflammatory blood markers and immunologic responses develop when acute-HCA sequentially progresses in EPM resulting in either PTL or preterm-PROM. However, previous studies show inconclusive results and, moreover, possessed limitations in terms of the diagnosis of the progression of acute-HCA in EPM, which did not evaluate the whole sub-divisions of EPM (i.e., decidua, the membranous trophoblast of chorion, the connective tissue of chorion, and amnion) as follows (Table 1): (1) not available for the diagnostic criteria of acute-HCA in EPM [19,23,27,30,32]; (2) does not include the decidua [17,18,19,22,23,27,30,32,34,36,37,39,42]; (3) does not include chorion [19,23,27,30,32,42]; (4) does not include the chorio-decidua [19,23,27,30,32,42]; (5) does not include the membranous trophoblast of chorion [19,23,27,30,32,42]; (6) does not include the connective tissue of chorion [19,23,27,30,32,42]; (7) does not divide chorio-decidua into chorion and decidua [15,19,20,21,23,27,28,29,30,32,38,40,42]; (8) does not divide chorion into membranous trophoblast and connective tissue [13,14,15,16,19,20,21,23,24,25,26,27,28,29,30,31,32,33,35,36,38,39,40,41,42]; (9) does not differentiate the connective tissue of the chorion from amnion [16,17,18,19,22,23,27,30,32,34,37]; (10) does not consider amniotropic neutrophils migration as a progression of acute-HCA in EPM [19,23,27,30,31,32,42].

### 4.3. The Usefulness of Neutrophil to Lymphocyte Ratio (NLR) as a Maternal Inflammatory Blood Marker during Pregnancy

What is noteworthy is that the absolute count of each neutrophil and lymphocyte, but not the percentage of each neutrophil and lymphocyte as a relative ratio within leukocytes, should be interpreted cautiously because leukocytosis usually occurs during normal pregnancy [66] and the normal range of leukocyte count is widely variable among pregnant women [67,68,69,70,71]. Therefore, it is reasonable that the percentage of each neutrophil and lymphocyte, but not the absolute count of each neutrophil and lymphocyte in maternal blood, is used for the differentiation between inflammation-free placenta and acute-HCA during antenatal period.

### 4.4. Biologic Plausibility about Increased Maternal Inflammatory Blood Markers According to the Progression of Acute Histologic Chorioamnionitis (Acute-HCA) in Extra-Placental Membranes (EPM)

We previously demonstrated that intra-amniotic infection and inflammation recruits maternal neutrophils to the feto-maternal interface of chorio-decidua from maternal decidual vessels in both preterm rhesus model and human spontaneous PTB [72]; moreover, intra-amniotic inflammatory responses are more severe according to outside-in neutrophils migration in the chorio-decidua of EPM in human spontaneous PTB (i.e., ‘inflammation restricted to decidua’, ‘inflammation restricted to the membranous trophoblast of chorion and the decidua’, and ‘inflammation in the connective tissue of chorion’) [12]. Given that ‘leukocyte integrin lymphocyte function-associated antigen 1 (LFA-1)’ and its endothelial ligand ‘intercellular adhesion molecule (ICAM)-1′ play an important role in the endothelial adhesivity and transmigration of neutrophils in the capillaries of in vivo and in vitro inflammation models [73,74], we should find evidence about the expression of LFA-1/ICAM-1 in both maternal blood and EPM in the context of acute-HCA to explain the biological plausibility with respect to the positive correlation between maternal NLRs and the progression of acute-HCA generated by outside-in neutrophils migration in EPM. Indeed, maternal blood ICAM-1 was reported to be a reliable indicator of acute-HCA among cases with either PTL [28,42] or preterm-PROM [42] in spite of the above-mentioned limitations in those studies [28,42]. Moreover, EPM shows about a five-fold elevation of LFA-1 and about a three-fold elevation of ICAM-1 in mRNA sequencing profiles in preterm rhesus macaques delivered after 48 h following intra-amniotic lipopolysaccharides (LPS) infusion in our previous study (unpublished data). Therefore, one can expect that maternal NLRs significantly and progressively increased according to the progression of acute-HCA generated by outside-in neutrophils migration in EPM.

### 4.5. Major Strengths and Limitation of This Study

Firstly, the current study analyzed the progression of acute-HCA in the whole sub-divisions of EPM (i.e., decidua, the membranous trophoblast of chorion, the connective tissue of chorion, and amnion). Secondly, this study demonstrated that increased maternal NLR is an independent risk factor for amnionitis, known as advanced acute-HCA in EPM, even after the adjustment for the potential confounding variables including GA at delivery. Thirdly, this study recommended maternal NLR as a maternal inflammatory blood maker for the identification of acute-HCA with the use of a simple and widely available CBC in every medical institution. Although we did not compare the specificity and sensitivity for the identification of amnionitis between maternal NLR and other tests such as cytokines and chemokines, the measurements of cytokines and chemokines are not generally and widely available in every hospital. Limitation of this study is that the positive and negative LRs of maternal NLR cut-off 7.75 for the identification of amnionitis remained low. However, we did not find any non-invasive maternal blood biomarker for amnionitis (Table S1) and, therefore, maternal NLR may be promising for future trials for the identification of amnionitis.

### 4.6. Significance of This Study

This is the first human research reporting that maternal NLRs are significantly and positively correlated with the progression of acute-HCA in the whole sub-divisions of EPM (Figure 4) and that maternal NLRs are an independent risk factor for amnionitis, known as advanced acute-HCA, even after the correction for the potential confounding variables. This finding suggests maternal NLR may be used as a non-invasive antenatal marker for amnionitis.

### 4.7. Unanswered Questions and Proposals for Future Study

It is not yet known whether maternal inflammatory blood markers (i.e., NLR) can be used for the prediction for early acute-HCA in EPM (i.e., inflammation restricted to the decidua and inflammation restricted to the membrane trophoblast of chorion). This kind of study will improve the value of non-invasive maternal blood inflammatory markers for the early identification of pregnant women at risk for spontaneous PTB. However, the evaluation of the performance of NLR should clearly require a prospective description of this parameter in a cohort of patients with threatened PTL or preterm-PROM, including a part of patients remaining undelivered as is observed in real life.

## 5. Conclusions

Maternal NLRs significantly and progressively increased according to the progression of acute-HCA and increased maternal NLR (≥7.75) was an independent risk factor for amnionitis in spontaneous PTB.

## Figures and Tables

**Table 1:** Clinical characteristics and pregnancy outcomes according to the progression of acute histologic chorioamnionitis (acute-HCA) in extra-placental membranes (EPM).

	Group-0 ^†^	Group-1 ^†^	Group-2 ^†^	Group-3 ^†^	Group-4 ^†^	*p* Value ^a^
	36.4% (48/132)	14.4% (19/132)	20.5% (27/132)	17.4% (23/132)	11.4% (15/132)	
Maternal age, year (mean ± SD)	32.8 ± 4.6	32.9 ± 3.4	32.5 ± 3.9	34.7 ± 3.6	33.2 ± 4.6	NS (0.302)
Nulliparity	50.0% (24/48)	47.4% (9/19)	37.0% (10/27)	34.8% (8/23)	33.3% (5/15)	NS (0.610)
Either clinical history of antenatal vaginal bleeding or evidence of placenta previa	18.8% (9/48)	15.8% (3/19)	0% (0/27)	4.3% (1/23)	13.3% (2/15)	NS (0.107)
Preterm-PROM as a cause of PTB	50.0% (24/48)	31.6% (6/19)	59.3% (16/27)	69.6% (16/23)	46.7% (7/15)	NS (0.145)
Male Newborn	58.3% (28/48)	57.9% (11/19)	70.4% (19/27)	34.8% (8/23)	66.7% (10/15)	NS (0.125)
Cesarean delivery	41.7% (20/48)	31.6% (6/19)	18.5% (5/27)	34.8% (8/23)	33.3% (5/15)	NS (0.378)
Median GA at delivery, weeks (range)	31.6 (21.6–33.9)	30.3 (23.4–33.7)	30.3 (20.6–33.4)	28.0 (22.0–31.9) ^b^	26.6 (21.3–31.4) ^c, d^	<0.001
Birth weight, g (mean ± SD)	1572 ± 567	1478 ± 547	1444 ± 636	1129 ± 330 ^e^	1057 ± 419 ^e^	0.003
1 min Apgar score of <7	77.1% (37/48)	63.2% (12/19)	66.7% (18/27)	91.3% (21/23)	80.0% (12/15)	NS (0.193)
5 min Apgar score of <7	31.2% (15/48)	26.3% (5/19)	22.2% (6/27)	43.5% (10/23)	60.0% (9/15)	NS (0.101)
Meconium staining	8.3% (4/48)	0% (0/19)	0% (0/27)	17.4% (4/23)	6.7% (1/15)	NS (0.108)
Antenatal use of corticosteroids	81.2% (39/48)	68.4% (13/19)	77.8% (21/27)	82.6% (19/23)	86.7% (13/15)	NS (0.702)
Antenatal use of antibiotics	70.8% (34/48)	73.7% (14/19)	81.5% (22/27)	100% (23/23) ^f^	93.3% (14/15)	0.029
Antenatal use of tocolytics	66.7% (32/48)	78.9% (15/19)	81.5% (22/27)	91.3% (21/23)	86.7% (13/15)	NS (0.145)

GA, gestational age; NS, not significant; preterm-PROM, preterm premature rupture of membranes; PTB, preterm birth; SD, standard deviation. ^†^ Group-0: inflammation-free extra-placental membranes (EPM). ^†^ Group-1: inflammation restricted to decidua. ^†^ Group-2: inflammation restricted to the membranous trophoblast of chorion and the decidua. ^†^ Group-3: inflammation in the connective tissue of chorion but not the amnion. ^†^ Group-4: amnionitis. ^a^ Intergroup difference by Chi-square test (categorical variables) and Kruskal–Wallis test (continuous variables). ^b^ *p* < 0.05 vs. group-0 (1-way ANOVA with post-hoc Tukey test). ^c^ *p* < 0.005 vs. group-0 (1-way ANOVA with post-hoc Tukey test). ^d^ *p* < 0.05 vs. group-1 (1-way ANOVA with post-hoc Tukey test). ^e^ *p* < 0.05 vs. group-0 (1-way ANOVA with post-hoc Tukey test). ^f^ *p* < 0.05 vs. group-0 (Fisher’s exact test with Bonferroni’s correction).

**Table 2:** Diagnostic indices, predictive values, and likelihood ratios of maternal NLR (neutrophil to lymphocyte ratio) ≥ 7.75 within 48 h before delivery for the identification of amnionitis in cases with either preterm labor and intact membranes (PTL) or preterm premature rupture of membranes (preterm-PROM) (The prevalence of amnionitis is 11.4% (15/132)).

	Sensitivity	Specificity	Positive Predictive Value	Negative Predictive Value	Positive LR (95% CI)	Negative LR (95% CI)
NLR ≥ 7.75	80.0% (12/15)	59.0% (69/117)	20.0% (12/60)	95.8% (69/72)	2.9487 (1.0597–8.2047)	0.5128 (0.3674–0.7158)

CI, confidence interval; LR, likelihood ratio; NLR, neutrophil to lymphocyte ratio.

**Table 3:** Relationship of various independent variables with amnionitis analyzed by overall logistic regression analysis.

	Odds Ratio	95% Confidence Interval	*p* Value
Increased maternal NLR (≥7.75)	5.559	1.255–24.621	0.024
Gestational age at delivery (on a daily basis)	0.716	0.562–0.912	0.007
Parity (≥1)	2.209	0.567–8.602	NS (0.253)
Preterm-PROM as a cause of PTB	1.258	0.311–5.091	NS (0.748)
Vaginal delivery	0.945	0.217–4.116	NS (0.940)
Antenatal corticosteroids use	9.474	0.989–90.794	NS (0.051)
Antenatal antibiotics use	0.980	0.076–12.607	NS (0.987)
Antenatal tocolytics use	1.367	0.193–9.665	NS (0.754)
Meconium staining	0.752	0.071–7.938	NS (0.813)
Male sex of newborn	1.765	0.486–6.406	NS (0.388)
Either clinical history of antenatal vaginal bleedingor the evidence of placenta previa	2.674	0.296–24.131	NS (0.381)

NLR, neutrophil to lymphocyte ratio; preterm-PROM, preterm premature rupture of membranes; PTB, preterm birth.

## Supplementary Materials

The following are available online at https://www.mdpi.com/article/10.3390/jcm10122673/s1, Figure S1: Maternal high sensitivity C-reactive protein (hs-CRP) (mg/dL) according to the progression of acute histologic chorioamnionitis (acute-HCA) in extra-placental membranes (EPM), Figure S2: A receiver operating characteristics (ROC) curve was constructed to select the cut-off values at which to identify maternal NLR, as being raised or not raised for the identification of amnionitis, Figure S3: Frequency of increased maternal high sensitivity C-reactive protein (hs-CRP) (≥1.035 mg/dL) according to the progression of acute histologic chorioamnionitis (acute-HCA) in extra-placental membranes (EPM), Table S1: Previous studies reporting the relationship between maternal inflammatory blood markers and acute histologic chorioamnionitis (acute-HCA) in ex-tra-placental membranes (EPM), Table S2: Diagnostic indices, predictive values, and likelihood ratios of maternal high sensitivity C-reactive protein (hs-CRP) ≥ 1.035 mg/dL within 48 h before delivery for the identification of amnionitis in cases with either preterm labor and intact membranes (PTL) or preterm premature rupture of membranes (preterm-PROM), Table S3: Relationship of various independent variables with amnionitis analyzed by overall logistic regression analysis.
