# Supplementary material for: Neutrophil to Lymphocyte Ratio in Maternal Blood: A Clue to Suspect Amnionitis
Source: J Clin Med. 2021 Jun 17;10(12):2673. doi: 10.3390/jcm10122673 (PMC8235298; doi:10.3390/jcm10122673)
Supplement: Supplementary file 1 [file jcm-10-02673-s001.zip › New Supplementary Table 1.pdf]

Supplementary Table S1. Previous studies reporting the relationship between maternal inflammatory blood markers and acute histologic chorioamnionitis (acute-HCA) in extra-placental membranes (EPM).

Primary author (year)	Inclusion Criteria (i.e., GAD)	Timing of maternal blood tests	Causes of PTB (N)	N (HCA [-] vs. HCA [+])	Diagnostic criteria for acute-HCA in EPM	Inflammation in the subdivisions of EPM	Inclusion of whole subdivision of EPM	Frequency of inflammation in subdivisions of EPM [%]	Maternal inflammatory blood marker	Correlation between each marker and acute-HCA in EPM		Correlation between each marker and the progression of acute-HCA in EPM	
										No adjustment	Adjustment for GAD	No adjustment	Adjustment for GAD
Kidokoro (2006) [13]	1. Singleton<br>2. GA: 16-35 wks<br>3. PTL, Preterm-PROM<br>4. Delivered <48 h after amniocentesis	Delivered <48 h after maternal blood test	Threatened abortion, PTL, preterm-PROM	60 (15 vs. 45)	Blanc's criteria [43]	Stage I : Intervillositis<br>Stage II: Chorionitis<br>Stage III: CAM	N/A	N/A	1. WBC<br>2. CRP	1. HCA (-) < HCA (+)<br>2. HCA (-) < HCA (+)	N/A	N/A	N/A
Erdemir (2013) [14]	GAD < 35 wks	During labor	N/A	43 (33 vs. 10)	Maternal neutrophils infiltration into amnion, chorion, and decidua	N/A	N/A	N/A	1. WBC<br>2. CRP	1. HCA (-) = HCA (+)<br>2. HCA (-) = HCA (+)	N/A	N/A	N/A
Park (2013) [15]	1. 20.0 ≤ GAD <34.5 wks<br>2. PTB due to PTL<br>3. IAI<br>4. Delivered <5 d of amniocentesis	Delivered <5 d after maternal blood test	PTL (53)	53 (7 vs. 42)	Modified Salafia's criteria [44]	Choriodeciduitis<br>Amnionitis	N/A	Chorio-deciduitis: 73.5%,<br>Amnionitis: 45.3%	1. CRP	1. HCA (-) < HCA (+)	N/A	Amnionitis; CRP<0.7 vs. CRP≥0.7 (mg/dL) =7.1% [1/14] ↓ vs. 50.9% [23/39] ↑	Anelevated CRP was predictor for amnionitis.
Kim (2014) [16]	1. Singleton<br>2. PTB due to preterm-PROM or PTL<br>3. GAD: 24-37 wks	On admission.	PTB (483): PTL (245) vs. preterm-PROM (238)	442 (196 vs. 246)	MIR: subchorionitis, CAM, deciduitis, or free membranitis	N/A	N/A	N/A	1. Neutrophil<br>2. Lymphocyte<br>3. Monocyte<br>4. Eosinophil<br>5. Basophil<br>6. CRP<br>7. NLR	1. HCA (-) < HCA (+)<br>2. HCA (-) < HCA (+)<br>3. HCA (-) < HCA (+)<br>4. HCA (-) = HCA (+)<br>5. HCA (-) = HCA (+)<br>6. HCA (-) < HCA (+)<br>7. HCA (-) < HCA (+)	N/A	N/A	N/A

Martinez-<br>Portilla<br>(2019)<br>[17]	1. Singleton<br>2. GAD: 30-35 wks<br>3. Preterm-PROM	IL-6: at<br>enrollment;<br>WBC: every 72 h	Preterm-PROM (47)	47<br>(31 vs. 16)	Redline's criteria<br>[6]	Stage 1: Acute<br>subchorionitis or<br>chorionitis<br>Stage 2: ACAM<br>Stage 3: necrotizing CAM	N/A	N/A	1. WBC<br>2. Leukocytosis<br>3. Segmented<br>cells<br>4. IL-6 (pg/mL)	1. HCA (-) = HCA (+)<br>2. HCA (-) = HCA (+)<br>3. HCA (-) = HCA (+)<br>4. HCA (-) < HCA (+)	N/A	N/A	N/A
Howman<br>(2012)<br>[18]	GAD: 24-42 wks	Immediately<br>prior to delivery	N/A<br>* PTB (222)	325<br>(299 vs. 26)	Redline's criteria<br>[6]	Stage 1: Acute<br>subchorionitis or<br>chorionitis<br>Stage 2: ACAM<br>Stage 3: necrotizing CAM	N/A	Stage 1: 23.1%<br>Stage 2: 34.6%<br>Stage 3: 42.3%	1. hsCRP (mg/L)<br>2. Procalcitonin	1. HCA (-) < HCA (+)<br>2. HCA (-) = HCA (+)	N/A	N/A	N/A
Xie<br>(2015)<br>[19]	1. preterm-PROM < 34 wks	At admission<br>and before<br>delivery	Preterm-PROM<br>(371)	371<br>(110 vs.<br>261)	N/A	N/A	N/A	N/A	1. CRP<br>2. WBC	Before delivery<br>HCA (-) < HCA (+)<br>Before delivery<br>HCA (-) = HCA (+)	N/A	N/A	N/A
Kim<br>(2016)<br>[20]	1. Singleton<br>2. GA 20-34 wks	At amniocentesis	Preterm-PROM<br>(181)	146<br>(72 vs. 74)	Modified<br>Salafia's criteria<br>[44]	Choriodeciduitis<br>Amnionitis	N/A	N/A	1. WBC<br>2. CRP	1. HCA (-) < HCA (+)<br>2. HCA (-) < HCA (+)	N/A	N/A	N/A
Park<br>(2019)<br>[21]	1. PTL 23-34 weeks<br>2. Delivered <96 h of<br>maternal blood test	Delivered <96 h<br>of blood test	PTL (73)	73<br>(30 vs. 44)	Modified<br>Salafia's criteria<br>[44]	Choriodeciduitis<br>Amnionitis	N/A	N/A	1. CRP<br>2. MMP-9<br>3. TIMP-1<br>4. IL-6<br>5. Angiopoietin-2<br>6. S100 A8/A9<br>7. CXCL14<br>8. APRIL<br>9. IGFBP-2	1. HCA (-) < HCA (+)<br>2. HCA (-) = HCA (+)<br>3. HCA (-) = HCA (+)<br>4. HCA (-) < HCA (+)<br>5. HCA (-) = HCA (+)<br>6. HCA (-) < HCA (+)<br>7. HCA (-) = HCA (+)<br>8. HCA (-) = HCA (+)<br>9. HCA (-) < HCA (+)	N/A	N/A	N/A
Hackney<br>(2008)<br>[22]	1. Singleton<br>2. Before 13 wks of<br>estimated GA	At enrollment	PTB (8)<br>HCA (-) vs. HCA (+)<br>= 8.3% vs. 13.9%	Nested-<br>case<br>control<br>study<br>72	Redline's criteria<br>[6]	Stage 1: Acute<br>subchorionitis or<br>chorionitis<br>Stage 2: ACAM<br>Stage 3: necrotizing CAM	N/A	Stage 1: 58%<br>Grade 1: 69%	CRP	HCA (-) = HCA (+)	HCA (-) =<br>HCA (+)	N/A	N/A

	3. GAD: HCA (-), 38.3 [34.2-41.2] wks; HCA (+), 38.3 [17-41] wks			(36 vs. 36)									
Wu (2009) [23]	1. Preterm infants: NICU admission<br>2. GAD: HCA (-), 33.0±2.6 wks; HCA (+), 30.8±4.1 wks	N/A	N/A	77 (50 vs. 27)	Diffuse infiltration or aggregation of PMN leukocytes in chorioamnion plate.	N/A	N/A	N/A	1. WBC<br>2. Segments<br>3. Lymphocytes<br>4. Bands<br>5. CRP<br>6. Hb<br>7. Platelets	1. HCA (-) < HCA (+)<br>2. HCA (-) = HCA (+)<br>3. HCA (-) = HCA (+)<br>4. HCA (-) = HCA (+)<br>5. HCA (-) = HCA (+)<br>6. HCA (-) = HCA (+)<br>7. HCA (-) = HCA (+)	N/A	N/A	N/A
Yamada (2000) [24]	1. Singleton<br>2. Intact membranes without bleeding<br>3. GAD, 27-41 wks	N/A	PTL (6)	39 (29 vs. 10)	Blanc's criteria [43]	Stage I: Intervillositis<br>Stage II: Chorionitis<br>Stage III: CAM	N/A	N/A	1. WBC<br>2. CRP	1. HCA (-) = HCA (+)<br>2. HCA (-) = HCA (+)	N/A	N/A	N/A
Kurakazu (2019) [25]	1. Singleton<br>2. PTL<br>3. suspected intrauterine infection<br>4. GAD: HCA (-), 34.3±4.3 wks; HCA (+), 29.3±3.7 wks	N/A	PTL (80)	80 (31 vs. 49)	Blanc's criteria [43]	Stage I: Intervillositis<br>Stage II: Chorionitis<br>Stage III: CAM	N/A	N/A	1. WBC<br>2. CRP	1. HCA (-) < HCA (+)<br>2. HCA (-) < HCA (+)	N/A	N/A	N/A
Maeda (1997) [26]	1. GAD 22-34 wks	At delivery	PTB (29) = PTL vs Preterm-PROM	29 (11 vs. 18)	Blanc's criteria [45]	Stage I: Intervillositis<br>Stage II: Chorionitis<br>Stage III: CAM	N/A	Stage 1: 5.6%<br>Stage 2: 55.6%<br>Stage 3: 38.9%	1. IL-6<br>2. IL-8<br>3. MCAF<br>4. CRP<br>5. WBC<br>6. sIL-6R	1. HCA (-) < HCA (+)<br>2. HCA (-) = HCA (+)<br>3. HCA (-) = HCA (+)<br>4. HCA (-) < HCA (+)<br>5. HCA (-) = HCA (+)<br>6. HCA (-) = HCA (+)	N/A	N/A	N/A
Kwak (2015) [27]	1. PTL<br>2. Preterm-PROM<br>3. GAD, 34.1±3.7 wks	At admission	PTB (165) = PTL (90) vs. PROM (75)	165 (121 vs. 45)	N/A	N/A	N/A	N/A	1. CRP	1. HCA (-) < HCA (+)	N/A	N/A	N/A

Steinborn<br>(2000)<br>[28]	1. Singleton<br>2. GAD, 13-36 wks	Delivered <72 h<br>of blood test	PTB (PTL<br>vs.preterm- PROM)<br>97 (36 vs. 61)	97<br>(49 vs. 48)	Modified<br>Salafia's criteria<br>[44]	Choriodeciduitis<br>Amnionitis	N/A	N/A	1. ICAM-1<br>2. WBC<br>3. CRP	1. HCA (-) < HCA (+)<br>2. HCA (-) < HCA (+)<br>3. HCA (-) < HCA (+)	N/A	N/A	N/A
Oh<br>(2011)<br>[29]	1. Singleton<br>2. PTL or preterm-PROM<br>2. GA 21-35 wks<br>3. Delivery <72 h of<br>amniocentesis	At amniocentesis	PTB (99)<br>= PTL (38) vs.<br>preterm-PROM (61)	99<br>(55 vs. 44)	Modified<br>Salafia's criteria<br>[44]	Choriodeciduitis<br>Amnionitis	N/A	N/A	1. CRP	1. HCA (-) < HCA (+)	N/A	N/A	N/A
Makino<br>(2003)<br>[30]	1. Singleton<br>2. GAD 24-39 wks	Before delivery	N/A	18<br>(10 vs. 8)	N/A	N/A	N/A	N/A	1. Total AM<br>2. Mature AM<br>3. CRP	1. HCA (-) < HCA (+)<br>2. HCA (-) < HCA (+)<br>3. HCA (-) < HCA (+)	N/A	N/A	N/A
Cho<br>(2017)<br>[31]	1. Singleton<br>2. GA 20-37 wks<br>3. Preterm-PROM	N/A	Preterm-PROM<br>(149)	149<br>(87 vs. 62)	N/A	N/A	N/A	Chorionitis:<br>11.3%,<br>Deciduitis:<br>11.3%,<br>Amnionitis<br>+ deciduitis:<br>3.2%	1. Hb<br>2. Hematocrit<br>3. Platelet<br>4. WBC<br>5. Neutrophil<br>6. Lymphocyte<br>7. Monocyte<br>8. Eosinophil<br>9. Basophil<br>10. PT<br>11. PTT<br>12. NLR<br>13. DNI<br>14. CRP	1. HCA (-) < HCA (+)<br>2. HCA (-) < HCA (+)<br>3. HCA (-) = HCA (+)<br>4. HCA (-) = HCA (+)<br>5. HCA (-) = HCA (+)<br>6. HCA (-) < HCA (+)<br>7. HCA (-) = HCA (+)<br>8. HCA (-) = HCA (+)<br>9. HCA (-) = HCA (+)<br>10. HCA (-) < HCA (+)<br>11. HCA (-) = HCA (+)<br>12. HCA (-) < HCA (+)<br>13. HCA (-) < HCA (+)<br>14. HCA (-) < HCA (+)	N/A	N/A	N/A
Broumand<br>(2018)<br>[32]	1. GAD, 29^{+5}-34^{+0} wks<br>2. Preterm-PROM	On admission	Preterm-PROM (48)	48<br>(29 vs. 19)	Leukocyte<br>infiltration with<br>neutrophilic<br>priority on fetal<br>membranes	N/A	N/A	N/A	1. WBC<br>2. ESR	1. HCA (-) < HCA (+)<br>2. HCA (-) < HCA (+)	N/A	N/A	N/A

Shimoya<br>(1997)<br>[33]	1. Spontaneous labor at term	At delivery	N/A	103<br>(81 vs. 22)	Blanc's criteria<br>[45]	Stage I: Intervillositis<br>Stage II: Chorionitis<br>Stage III: CAM	N/A	Stage 1: 72.7%<br>Stage 2: 18.2%<br>Stage 3: 9.1%	1. IL-1α<br>2. IL-1β<br>3. TNF-α<br>4. IL-6<br>5. IL-8	1. HCA (-) = HCA (+)<br>2. HCA (-) < HCA (+)<br>3. HCA (-) = HCA (+)<br>4. HCA (-) = HCA (+)<br>5. HCA (-) < HCA (+)	N/A	N/A	N/A
Ahmed<br>(2016)<br>[34]	1. Singleton<br>2. GA 24-34 wks<br>3. Preterm-PROM	On admission and delivery	Preterm-PROM (60)	60<br>(24 vs. 36)	Redline's criteria<br>[6]	Stage 1: Acute subchorionitis or chorionitis<br>Stage 2: ACAM<br>Stage 3: necrotizing CAM	N/A	N/A	1. IL-6	1. HCA (-) < HCA (+)	N/A	N/A	N/A
Yoneda<br>(2015)<br>[35]	1. C/S<br>2. GAD: HCA (-), 37wks [24-41]; HCA (+), 33wks [24-41]<br>3. Preterm-PROM(-)<br>4. Failure of trial of labor(-)	In preterm delivery <48 h before C/S,<br>In term <2 wks before C/S	N/A	428<br>(234 vs. 194)	Blanc's criteria<br>[43]	Stage I: Intervillositis<br>Stage II: Chorionitis<br>Stage III: CAM	N/A	Stage I: 47.4%<br>Stage II: 35.0%<br>Stage III: 17.6%	1. WBC<br>2. CRP	1. HCA (-) < HCA (+)<br>2. HCA (-) < HCA (+)	N/A	N/A	N/A
Gulati<br>(2012)<br>[36]	1. GA 24-34 wks<br>2. Preterm-PROM	On admission, at 48 h, and on day 7 of admission or onset of labor	Preterm-PROM (45)	45<br>(23 vs. 22)	Leucocytic infiltration in amnion, chorion, umbilical vessels	N/A	N/A	N/A	1. IL-6	1. HCA (-) < HCA (+)	N/A	N/A	N/A
Caloone<br>(2016)<br>[37]	1. Preterm-PROM<br>2. GA 22-37 wks	Every 48 h during hospitalization and at delivery	Preterm-PROM<br>(295)	295<br>(169 vs. 126)	Redline's criteria<br>[6]	Stage 1: Acute subchorionitis or chorionitis<br>Stage 2: ACAM<br>Stage 3: necrotizing CAM	N/A	Stage 1: 28.9%<br>Stage 2: 44.4%<br>Stage 3: 26.7%	1. IL-6<br>2. IL-8<br>3. HNP<br>4. TREM-1<br>5. MMP-8<br>6. MMP-9<br>7. ICAM-1<br>8. CRP	1. HCA (-) < HCA (+)<br>2. HCA (-) = HCA (+)<br>3. HCA (-) < HCA (+)<br>4. HCA (-) = HCA (+)<br>5. HCA (-) < HCA (+)<br>6. HCA (-) < HCA (+)<br>7. HCA (-) = HCA (+)<br>8. HCA (-) < HCA (+)	N/A	N/A	N/A
Cobo<br>(2012)<br>[38]	1. Singleton<br>2. GA 23-37 wks	On admission and every day until delivery	Preterm-PROM<br>(107)	107<br>(46 vs. 61)	Salafia's criteria£ [46]	Choriodeciduitis<br>Amnionitis	N/A	N/A	1. WBC<br>2. CRP	1. HCA (-) = HCA (+)<br>2. HCA (-) < HCA (+)	N/A	N/A	N/A

	3. Preterm-PROM												
Gulati<br>(2012)<br>[39]	1. Preterm-PROM<br>2. GA 24-34 wks<br>3. Singleton	At admission, at 48 h, and on day 7 of admission or onset of labor	Preterm-PROM (45)	45<br>(23 vs. 22)	Leucocytic infiltration in amnion, chorion, umbilical vessels	N/A	N/A	N/A	1. IL-6	1. HCA (-) < HCA (+)	N/A	N/A	N/A
Škrablin<br>(2007)<br>[40]	1. PTL<br>2. Preterm-PROM<br>3. GA 27-33 wks<br>4. GAD <37 wks	On admission & active phase of labor	PTB (47)<br>= PTL (20) vs.<br>Preterm-PROM (27)	47<br>(24 vs. 23)	Modified Salafia's criteria<br>[44]	Choriodeciduitis<br>Amnionitis	N/A	N/A	1. IL-6<br>2. IL-1β<br>3. hsCRP	1. HCA (-) < HCA (+)<br>2. HCA (-) < HCA (+)<br>3. HCA (-) < HCA (+)	N/A	N/A	N/A
Ohyama<br>(2002)<br>[41]	1. Singleton<br>2. GAD 23-32 wks	N/A	Preterm-PROM (90)	143<br>(53 vs. 90)	Blanc's criteria<br>[43]	Stage I: Intervillositis<br>Stage II: Chorionitis<br>Stage III: CAM	N/A	N/A	1. WBC<br>2. CRP	1. WBC<br>HCA (-) < ACAM (+)<br>HCA (-) < SCAM (+)<br>2. CRP<br>ACAM (+) < SCAM (+)<br>HCA (-) < ACAM (+)<br>HCA (-) < SCAM (+)	N/A	N/A	N/A
Zou<br>(2004)<br>[42]	1. Singleton	24 h prior to onset of labor or C/S	PROM (55)	55<br>(34 vs. 21)	PMN leukocyte infiltration of chorionic plate and amnion	N/A	N/A	N/A	1. ICAM-1<br>2. CRP	1. HCA (-) < HCA (+)<br>2. HCA (-) < HCA (+)	N/A	N/A	N/A

ACAM, acute chorioamnionitis; *AF,* amniotic fluid; *AM,* adrenomedullin; *APRIL,* a proliferation-inducing ligand; *CAM,* chorioamnionitis; *CRP,* C-reactive protein; *C/S,* cesarean section; *CXCL,* C-X-C motif chemokine ligand; *DNI,* delta neutrophil index; *EPM,* extraplacental membranes; *ESR,* erythrocyte sedimentation rate; *GA,* gestational age; *GAA,* gestational age at amniocentesis; *GAD,* gestational age at delivery; *Hb,* hemoglobin; *HCA,* histologic chorioamnionitis; *HNP,* human neutrophil peptides; *IAI,* intra-amniotic inflammation; *ICAM,* intercellular adhesion molecule; *IGFBP,* insulin-like growth factor-binding protein; *IL,* interleukin; *MCAF,* monocyte chemotactic and activating factor; *MIR,* maternal inflammatory response; *MMP,* matrix metalloproteinase; *N/A,* not available; *NICU,* neonatal intensive care unit; *NLR,* neutrophil to lymphocyte ratio; *PMN,* polymorphonuclear neutrophil; *preterm-PROM,* preterm premature rupture of membranes; *PT (INR),* prothrombin time (international normalized ratio); *PTB,* preterm birth; *PTL,* preterm labor and intact membranes; *PTT (sec),* partial thromboplastin time (seconds); *S100 A8/A9,* S100 calcium-binding protein A8/A9 complex; *SCAM,* subacute chorioamnionitis; *SD,* standard deviation; *sIL-6R,* soluble IL-6 receptor; *TIMP,* tissue inhibitor of metalloproteinase; *TNF,* tumor necrosis factor; *TREM,* triggering receptor of myeloid cells; *vs.,* versus; *WBC,* white blood cell.
