# Supplementary material for: Neutrophil to Lymphocyte Ratio in Maternal Blood: A Clue to Suspect Amnionitis
Source: J Clin Med. 2021 Jun 17;10(12):2673. doi: 10.3390/jcm10122673 (PMC8235298; doi:10.3390/jcm10122673)
Supplement: Supplementary file 1 [file jcm-10-02673-s001.zip › New Supplementary Table 2 and Table 3.pdf]

Supplementary Tables

Table S2. Diagnostic indices, predictive values, and likelihood ratios of maternal high sensitivity C-reactive protein (hs-CRP) ≥ 1.035 mg/dL within 48 hours before delivery for the identification of amnionitis in cases with either preterm labor and intact membranes (PTL) or preterm premature rupture of membranes (preterm-PROM) (The prevalence of amnionitis is 11.6% [14/121†]).

	Sensitivity	Specificity	Positive predictive value	Negative predictive value	Positive LR (95% CI)	Negative LR (95% CI)
hs-CRP ≥ 1.035 mg/dL	57.1% (8/14)	63.6% (68/108)	17.0% (8/47)	91.9% (68/74)	1.1074 [0.9569-1.2816]	0.4764 [0.1764-1.2863]

CI, confidence interval; *LR*, likelihood ratio; *hs-CRP*, High sensitivity C-reactive protein

† Among 132 patients included in this study, hs-CRP results within 48 hours before delivery were not available in 11 cases, and therefore, we analyzed hs-CRP data in only 121 patients.

Table S3. Relationship of various independent variables with amnionitis analyzed by overall logistic regression analysis†.

	Odds ratio	95% Confidence Interval	P value
Increased maternal hs-CRP (≥ 1.035 mg/dL)	2.996	0.777-11.553	NS (0.111)
Gestational age at delivery (on a daily basis)	0.789	0.633-0.985	0.036
Parity (≥ 1)	1.733	0.462-6.494	NS (0.415)
Preterm-PROM as a cause of PTB	1.463	0.391-5.472	NS (0.572)
Vaginal delivery	1.017	0.246-4.208	NS (0.981)
Antenatal corticosteroids use	2.100	0.298-14.790	NS (0.456)
Antenatal antibiotics use	2.207	0.167-24.566	NS (0.579)
Antenatal tocolytics use	1.945	0.313-12.081	NS (0.475)
Meconium staining	0.564	0.056-5.643	NS (0.626)
Male sex of newborn	1.283	0.367-4.486	NS (0.696)
Either clinical history of antenatal vaginal bleeding or the evidence of placenta previa	9.113	1.061-78.274	0.044

hs-CRP, high sensitivity C-reactive protein; *preterm-PROM,* preterm premature rupture of membranes; *PTB,* preterm birth.

† Among 132 patients included in this study, hs-CRP results within 48 hours before delivery were not available in 11 cases, and therefore, we analyzed hs-CRP data in only 121 patients.
