# Supplementary material for: Neutrophil to Lymphocyte Ratio in Maternal Blood: A Clue to Suspect Amnionitis
Source: J Clin Med. 2021 Jun 17;10(12):2673. doi: 10.3390/jcm10122673 (PMC8235298; doi:10.3390/jcm10122673)
Supplement: Supplementary file 1 [file jcm-10-02673-s001.zip › New Supplementary_Material_Figure legends.pdf]

Supplementary Materials

Supplementary Figure S1 (Figure S1). Maternal high sensitivity C-reactive protein (hs-CRP) (mg/dL) according to the progression of acute histologic chorioamnionitis (acute-HCA) in extra-placental membranes (EPM). Maternal hs-CRP (mg/dL) significantly and progressively increased with the progression of acute-HCA (group-0 vs. group-1 vs. group-2 vs. group-3 vs. group-4; median, range; 0.40 [0.01-5.69] vs. 0.33 [0.01-14.56] vs. 0.95 [0.12-7.07] vs. 1.26 [0.1-5.10] vs. 1.06 [0.1-9.45]). Each P value is shown in the graph. Among 132 patients included in this study, hs-CRP results within 48 hours before delivery were not available in 11 cases, and therefore, we analyzed hs-CRP data in only 121 patients.

Supplementary Figure S2 (Figure S2). A receiver operating characteristics (ROC) curve was constructed to select the cut-off values at which to identify maternal NLR (red line) (area under curve [AUC], 0.745; standard error [SE], 0.066; P=0.002), as being raised or not raised for the identification of amnionitis, and a cut-off value of 7.75 was chosen. Moreover, for the comparison with maternal NLR, we constructed a ROC curve to choose the cut-off values at which to discover maternal high sensitivity C-reactive protein (hs-CRP) (blue line) (AUC, 0.581; SE, 0.086; P=0.323), as being raised or not raised for the diagnosis of amnionitis, and a cut-off value of 1.035 mg/dL was chosen. Among 132 patients included in this study, hs-CRP results within 48 hours before delivery were not available in 11 cases, and therefore, we analyzed hs-CRP data in only 121 patients.

Supplementary Figure S3 (Figure S3). Frequency of increased maternal high sensitivity C-reactive protein (hs-CRP) (≥ 1.035 mg/dL) according to the progression of acute histologic chorioamnionitis (acute-HCA) in extra-placental membranes (EPM). Each P value is shown in the graph. Among 132 patients included in this study, hs-CRP results within 48 hours before delivery were not available in 11 cases, and therefore, we analyzed hs-CRP data in only 121 patients.
