# Supplementary figures and images for: Neutrophil to Lymphocyte Ratio in Maternal Blood: A Clue to Suspect Amnionitis
Source: J Clin Med. 2021 Jun 17;10(12):2673. doi: 10.3390/jcm10122673 (PMC8235298; doi:10.3390/jcm10122673)

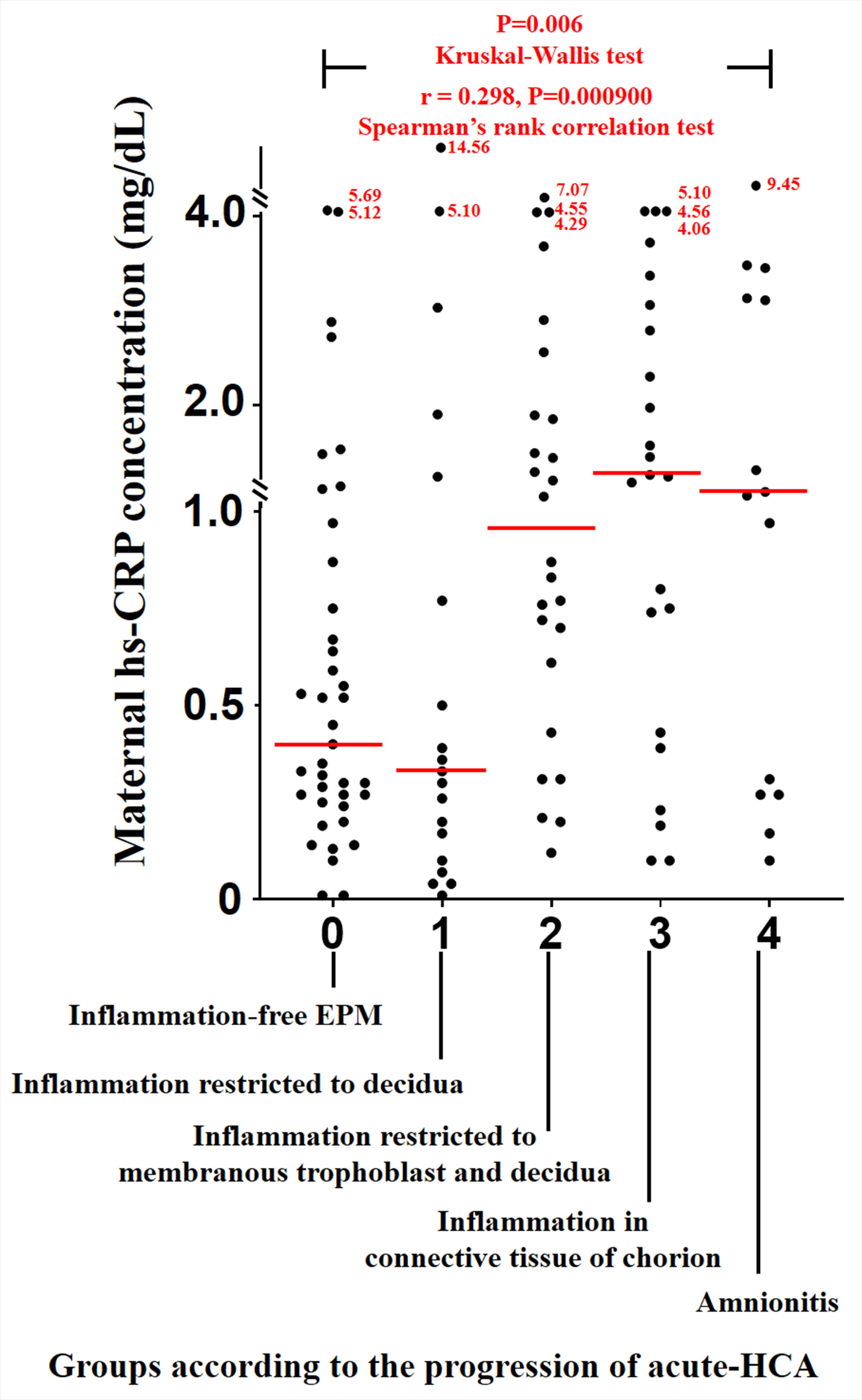

Supplement: Supplementary file 1 [file jcm-10-02673-s001.zip › New Supplementary Figure 1_300dpi.tif]

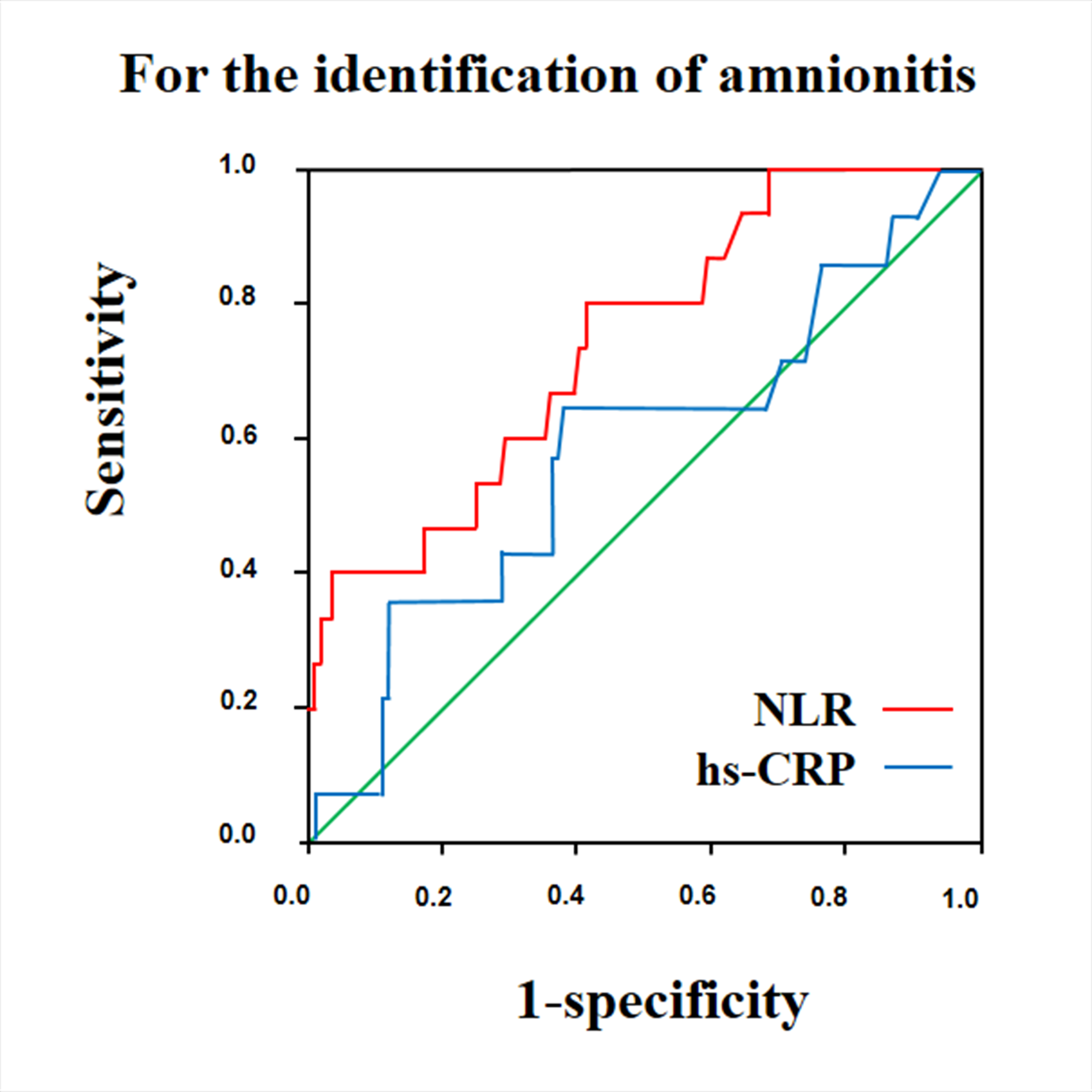

Supplement: Supplementary file 1 [file jcm-10-02673-s001.zip › New Supplementary Figure 2_300dpi.tif]

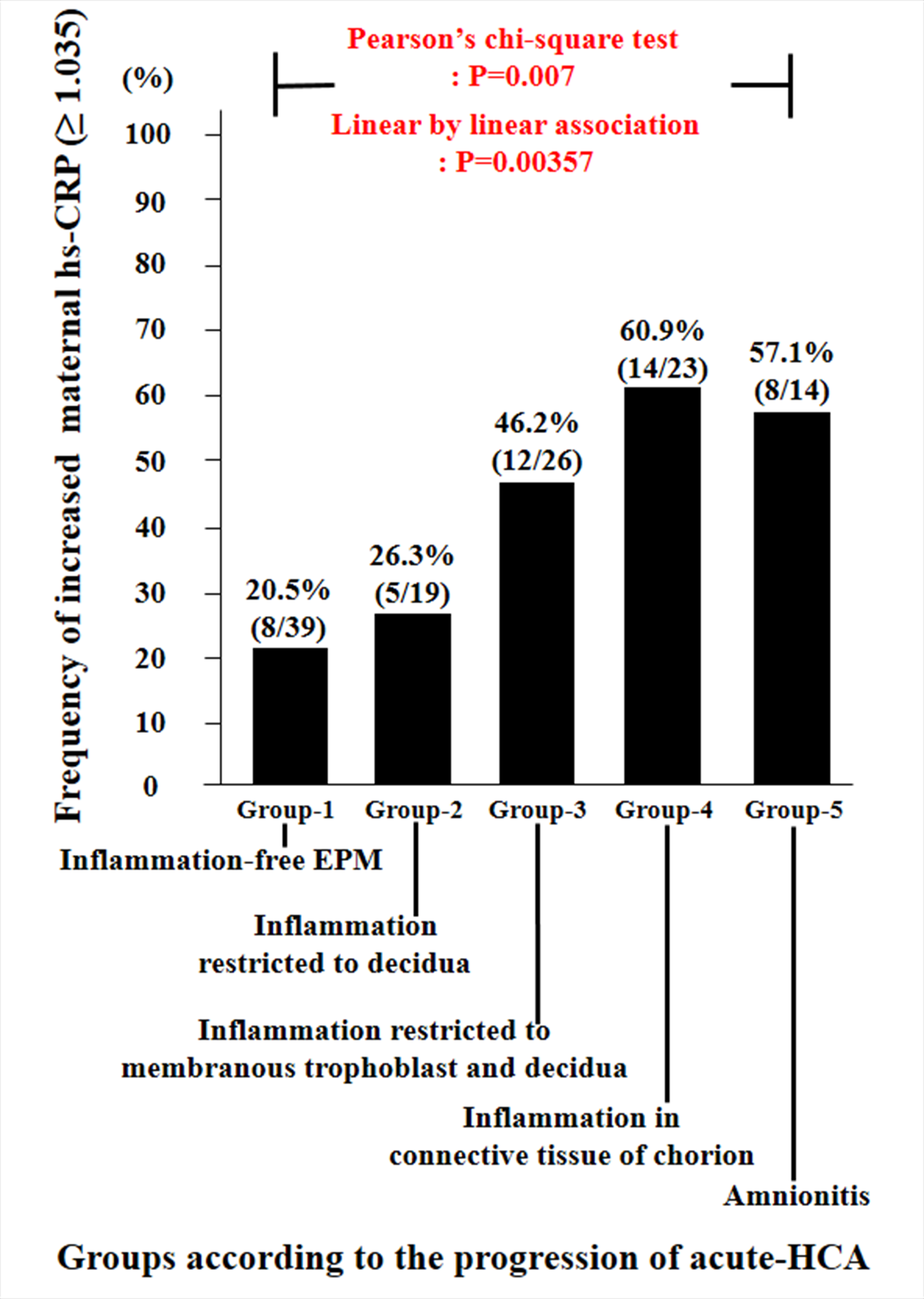

Supplement: Supplementary file 1 [file jcm-10-02673-s001.zip › New Supplementary Figure 3_300dpi.tif]
